# Supplementary material for: Development of the Ward Nurses’ Perspective-taking of the Staff Receiving Discharged Patients Scale: An observational study of ward nurses
Source: PLoS One. 2024 Nov 8;19(11):e0309883. doi: 10.1371/journal.pone.0309883 (PMC11548840; doi:10.1371/journal.pone.0309883)
Supplement: S1 Checklist — (DOCX) [file pone.0309883.s001.docx]

STROBE Statement—checklist of items that should be included in reports of observational studies

|  | Item No. | Recommendation | Page  No. | Relevant text from manuscript |
| --- | --- | --- | --- | --- |
| **Title and abstract** | 1 | (*a*) Indicate the study’s design with a commonly used term in the title or the abstract | 5 | **An Exploratory Study** |
|  |  | (*b*) Provide in the abstract an informative and balanced summary of what was done and what was found | 5 | This scale can measure the degree of perspective-taking by nurses, which can improve between-facility collaboration and the effectiveness of discharge planning. |
| Introduction | | | |  |
| Background/rationale | 2 | Explain the scientific background and rationale for the investigation being reported | 9 | While several measures of perspective-taking exist, none of these measure perspective-taking for staff receiving discharged patients [12,17–19].. |
| Objectives | 3 | State specific objectives, including any prespecified hypotheses | 9-10 | the aim of this study is to develop a scale to measure ward nurses’ perspective-taking of the staff receiving discharged patients (Ward Nurses’ Perspective-taking of the Staff Receiving Discharged Patients Scale ) and test the scale’s reliability and validity. |
| Methods | | | |  |
| Study design | 4 | Present key elements of study design early in the paper | 10, 13 | This study followed Boateng et al.’s [22] three steps of scale development: item development (literature review and interview), expert evaluation, and scale validation.  An online cross-sectional study was conducted from October to November 2021. |
| Setting | 5 | Describe the setting, locations, and relevant dates, including periods of recruitment, exposure, follow-up, and data collection | 13-14 | An online cross-sectional study was conducted from October to November 2021.  Overall, 45 facilities that agreed to participate were selected through random sampling of acute care hospitals without medical care beds in the Kanto Koshinetsu, Tohoku, and Tokai Hokuriku Health and Welfare Bureaus areas (selected based on facility reports issued by the Kanto-Shinetsu Regional Bureau of Health and Welfare [34], Tohoku Regional Bureau of Health and Welfare [35], and Tokai-Hokuriku Regional Bureau of Health and Welfare [36]) in Japan). |
| Participants | 6 | (*a*) *Cohort study*—Give the eligibility criteria, and the sources and methods of selection of participants. Describe methods of follow-up  *Case-control study*—Give the eligibility criteria, and the sources and methods of case ascertainment and control selection. Give the rationale for the choice of cases and controls  *Cross-sectional study*—Give the eligibility criteria, and the sources and methods of selection of participants | 14 | The inclusion criteria are ward nurses who had engaged in discharge planning. The exclusion criteria are nurses with less than a year of nursing experience, did not work in wards, worked in obstetric wards and unit care such as intensive care units and emergency wards, and worked as head ward nursing managers. |
|  |  | (*b*) *Cohort study*—For matched studies, give matching criteria and number of exposed and unexposed  *Case-control study*—For matched studies, give matching criteria and the number of controls per case | Not Applicable |  |
| Variables | 7 | Clearly define all outcomes, exposures, predictors, potential confounders, and effect modifiers. Give diagnostic criteria, if applicable | 16-17 | The comparative fit index (CFI), root mean square error of approximation (RMSEA), and standardized root mean squared residual (SRMR) were used as the evaluation indices for the CFA.  Internal consistency was confirmed by Cronbach’s alpha.  The perspective-taking subscale of the Japanese version of the Interpersonal Reactivity Index, [11, 41] measures perspective-taking in daily life.  The self-created single item (named “comprehensive perspective-taking”) “I think about discharge support from the perspective of the receiving staff.”  The sub-concept of the Self-centeredness Scale, which is also known as “inadequate empathy for others,” was developed by Hirosawa et al. [42].  The ICC (2, 1) [44] was calculated to verify temporal stability. |
| Data sources/ measurement | 8* | For each variable of interest, give sources of data and details of methods of assessment (measurement). Describe comparability of assessment methods if there is more than one group | 16-17 | 1) The perspective-taking subscale of the Japanese version of the Interpersonal Reactivity Index [11, 41] measures perspective-taking in daily life and was, therefore, a similar but slightly different concept from the Ward Nurses’ Perspective-taking of the Staff Receiving Discharged Patients Scale. The correlation was predicted to be weak to moderate. 2) Since there was no golden standard scale to measure comprehensive perspective-taking in discharge planning, we predicted that the Ward Nurses’ Perspective-taking of the Staff Receiving Discharged Patients Scale would have a moderate or high correlation with the self-created single item (named “comprehensive perspective-taking”) “I think about discharge support from the perspective of the receiving staff.” The participants were asked to respond to this statement on a 10-point scale ranging from 1= *not at all true* to 10 = *very true*. 3) The sub-concept of the Self-centeredness Scale, which is also known as “inadequate empathy for others,” was developed by Hirosawa et al. [42] based on Piaget’s [43] concept of self-centeredness. A negative correlation between this subconcept and perspective-taking was predicted. |
| Bias | 9 | Describe any efforts to address potential sources of bias | 14 | The exclusion criteria are nurses with less than a year of nursing experience, did not work in wards, worked in obstetric wards and unit care such as intensive care units and emergency wards, and worked as head ward nursing managers. |
| Study size | 10 | Explain how the study size was arrived at | 13 | We performed sample size calculations to validate construct validity at time 1 and temporal stability at time 2. Regarding time 1, we found that 200 participants, 10 times the number of items in the draft scale, were needed based on the recommendation of Boateng et al. [22]. Regarding time 2, we found 19 participants were needed to calculate the intraclass correlation coefficients (ICC) between the first and the second surveys with 30 observed variables per participant (the null hypothesis was set at 0.5, the alternative hypothesis at 0.7, the power at 0.8, and the probability of significance at 0.05) [31]. |

Continued on next page

| Quantitative variables | 11 | Explain how quantitative variables were handled in the analyses. If applicable, describe which groupings were chosen and why | 15-17 | To test for cross-validity, the collected sample was randomly divided into two groups. EFA was conducted for one group and confirmatory factor analysis (CFA) for the other. Exploratory factor analysis ascertained the number of factors that the items had been divided into, and CFA ascertained the goodness of fit of the factor structure detected by EFA. In the EFA, the number of factors was determined by scree plotting; subsequently, Promax rotation and unweighted least squares were selected. Since a Shapiro–Wilk test of the items did not confirm normality, the unweighted least squares method was selected. The comparative fit index (CFI), root mean square error of approximation (RMSEA), and standardized root mean squared residual (SRMR) were used as the evaluation indices for the CFA.  Internal consistency was confirmed by Cronbach’s alpha.  Construct validity was verified by calculating correlations between the Ward Nurses’ Perspective-taking of the Staff Receiving Discharged Patients Scale and similar concepts [40].  ICC (2, 1) [44] was calculated to verify temporal stability. |
| --- | --- | --- | --- | --- |
| Statistical methods | 12 | (*a*) Describe all statistical methods, including those used to control for confounding | 15-17 | Items that were similar or did not adequately measure perspective-taking were removed based on the following criteria: 1) inter-item correlations greater than 0.60 [22]; 2) item total (IT) correlations less than 0.30 [22]; 3) factor loadings in the exploratory factor analysis (EFA) of 0.30 or greater for multiple factors [37]; 4) commonality less than 0.4 in the EFA [38]  To test for cross-validity, the collected sample was randomly divided into two groups. EFA was conducted for one group and confirmatory factor analysis (CFA) for the other. In the EFA, the number of factors was determined by scree plotting; subsequently, Promax rotation and unweighted least squares were selected. Since a Shapiro–Wilk test of the items did not confirm normality, the unweighted least squares method was selected. The comparative fit index (CFI), root mean square error of approximation (RMSEA), and standardized root mean squared residual (SRMR) were used as the evaluation indices for the CFA.  Internal consistency was confirmed by Cronbach’s alpha. The criterion for the index was set at 0.7 [39]  Construct validity was verified by calculating correlations between the Ward Nurses’ Perspective-taking of the Staff Receiving Discharged Patients Scale and similar concepts [40].  ICC (2, 1) [44] was calculated to verify temporal stability. |
|  |  | (*b*) Describe any methods used to examine subgroups and interactions | Not applicable |  |
|  |  | (*c*) Explain how missing data were addressed | 17 | The online survey did not have any missing data because participants were unable to submit the survey unless they answered all the questions. |
|  |  | (*d*) *Cohort study*—If applicable, explain how loss to follow-up was addressed  *Case-control study*—If applicable, explain how matching of cases and controls was addressed  *Cross-sectional study*—If applicable, describe analytical methods taking account of sampling strategy | Not applicable |  |
|  |  | (*e*) Describe any sensitivity analyses | Not applicable |  |
| Results | | | | |
| Participants | 13* | (a) Report numbers of individuals at each stage of study (e.g., numbers potentially eligible, examined for eligibility, confirmed eligible, included in the study, completing follow-up, and analyzed) | 17 | For the first survey (Time 1), the questionnaire was distributed to 1,289 participants, and 416 valid responses were obtained (Figure 1). For the second survey (time 2), questionnaires were distributed to 172 participants who participated in the first survey and offered to cooperate in the second, and 61 valid responses were obtained. |
|  |  | (b) Give reasons for non-participation at each stage | Figure 1 | ^b^ Since there were two responses with the same ID and matching basic attributes, we considered them as duplicates and adopted the latest response.  ^c^ Respondents who agreed to cooperate in the second survey (Time 2) from among those who participated in the first survey (Time 1).  Exclusion: 1 no ID support |
|  |  | (c) Consider use of a flow diagram | Figure 1 |  |
| Descriptive data | 14* | (a) Give characteristics of study participants (e.g., demographic, clinical, social) and information on exposures and potential confounders | 18 | In the sample surveyed at Time 1 (*n* = 416), 93.8% were women, the mean age was 38.90 years, and the mean clinical experience was 15.62 years. Of the total, 67.1% of the participants held a staff position, and 44.2% had participated in discharge planning at least twice a month in the previous year (Table 1). |
|  |  | (b) Indicate number of participants with missing data for each variable of interest | Not applicable |  |
|  |  | (c) *Cohort study*—Summarize follow-up time (e.g., average and total amount) | Not applicable |  |
| Outcome data | 15* | *Cohort study*—Report numbers of outcome events or summary measures over time | Not applicable |  |
|  |  | *Case-control study—*Report numbers in each exposure category, or summary measures of exposure | Not applicable |  |
|  |  | *Cross-sectional study—*Report numbers of outcome events or summary measures | 21-22 | The CFA of the factor structure validated by the EFA yielded a CFI of 0.95, an RMSEA of 0.08, and an SRMR of 0.06 (Figure 2). Cronbach’s alpha for the Ward Nurses’ Perspective-taking of the Staff Receiving Discharged Patients Scale, calculated as the sum of the means of the subscales, was 0.89. The correlation coefficients between the Ward Nurses’ Perspective-taking of the Staff Receiving Discharged Patients Scale and the Japanese version of the Interpersonal Reactivity Index subscales of “perspective-taking” (excluding reversal items), “inclusive perspective-taking,” and “inadequate empathy for others” were 0.38, 0.57, and -0.33, respectively (Table 3). |
| Main results | 16 | (*a*) Give unadjusted estimates and, if applicable, confounder-adjusted estimates and their precision (e.g., 95% confidence interval). Make clear which confounders were adjusted for and why they were included | 21-22 | The CFA of the factor structure validated by the EFA yielded a CFI of 0.95, an RMSEA of 0.08, and an SRMR of 0.06 (Figure 2). Cronbach’s alpha for the Ward Nurses’ Perspective-taking of the Staff Receiving Discharged Patients Scale, calculated as the sum of the means of the subscales, was 0.89. The correlation coefficients between the Ward Nurses’ Perspective-taking of the Staff Receiving Discharged Patients Scale and the Japanese version of the Interpersonal Reactivity Index subscales of “perspective-taking” (excluding reversal items), “inclusive perspective-taking,” and “inadequate empathy for others” were 0.38, 0.57, and -0.33, respectively (Table 3). |
|  |  | (*b*) Report category boundaries when continuous variables were categorized | Not applicable |  |
|  |  | (*c*) If relevant, consider translating estimates of relative risk into absolute risk for a meaningful time period | Not applicable |  |

Continued on next page

| Other analyses | 17 | Report other analyses done—(e.g., analyses of subgroups and interactions, and sensitivity analyses) | Not applicable |  |
| --- | --- | --- | --- | --- |
| Discussion | | | | |
| Key results | 18 | Summarize key results with reference to study objectives | 25 | The reliability and validity of the developed scale to measure ward nurses’ perspective-taking of the staff receiving discharged patients developed in this study were verified. |
| Limitations | 19 | Discuss limitations of the study, taking into account sources of potential bias or imprecision. Discuss both direction and magnitude of any potential bias | 29-30 | The current study was conducted with nurses in acute care hospitals in Japan, which affects the generalizability of its findings. The viability of the scale for nurses other than ward nurses, such as those involved in advanced discharge planning [56], needs to be examined further. The current study met the sample size requirements for time 1 and time 2 [22,31]. However, the response rate was low, similar to the response rate in a Japanese study of discharge planning. [32,33]. The low response rate may be due to the many questions and participants’ interest. In particular, a selection bias may have occurred if only nurses who expressed a strong interest in discharge planning were assumed to have responded to the study questions.  In the present study, the majority of the participants were female. It has been reported that gender does not affect perspective-taking [18]. Therefore, a bias due to gender is unlikely. However, perspective-taking is affected by the power relationship with the target [57,58] as well as differences between competitive and cooperative environments [16]. Therefore, future research should not only focus on the attributes of the participant's population, but also on the relationship between the perspective-taking target and participant population.  In addition, the imagine-self subscale showed low temporal stability; therefore, there is a limitation to using this subscale by itself. Future longitudinal studies are needed to understand the nature of this subscale and explore avenues to recognize temporal trends in its scores.  Perspective-taking does not question the accuracy of understanding the object’s perspective [12]. Consequently, the ideas derived through perspective-taking do not necessarily correspond to what the target individual thinks or the actual situation in which that individual is placed. Future studies should evaluate the relationship between perspective-taking and inter-facility collaboration in detail. To do so, researchers should ask staff receiving discharged patients to evaluate the discharge planning in which the perspective-taking was demonstrated, using the Ward Nurses’ Perspective-taking of the Staff Receiving Discharged Patients Scale. |
| Interpretation | 20 | Give a cautious overall interpretation of results considering objectives, limitations, multiplicity of analyses, results from similar studies, and other relevant evidence | 30 | The reliability and validity of the Ward Nurses’ Perspective-taking of the Staff Receiving Discharged Patients Scale and its subscales were also verified in this study. |
| Generalizability | 21 | Discuss the generalizability (external validity) of the study results | 29 | The viability of the scale for nurses other than ward nurses, such as those involved in advanced discharge planning [54], needs to be examined further. |
| Other information | |  | | |
| Funding | 22 | Give the source of funding and the role of the funders for the present study and, if applicable, for the original study on which the present article is based | 3 | This study was supported by a scholarship awarded to Shingo Tanaka from the Global Creative Leaders (GCL) program of the University of Tokyo by the Ministry of Education, Culture, Sports, Science and Technology (MEXT), Japan. |

*Give information separately for cases and controls in case-control studies and, if applicable, for exposed and unexposed groups in cohort and cross-sectional studies.

**Note:** An Explanation and Elaboration article discusses each checklist item and gives methodological background and published examples of transparent reporting. The STROBE checklist is best used in conjunction with this article (freely available on the Web sites of PLoS Medicine at http://www.plosmedicine.org/, Annals of Internal Medicine at http://www.annals.org/, and Epidemiology at http://www.epidem.com/). Information on the STROBE Initiative is available at www.strobe-statement.org.
