# Supplementary material for: Development of the Ward Nurses’ Perspective-taking of the Staff Receiving Discharged Patients Scale: An observational study of ward nurses
Source: PLoS One. 2024 Nov 8;19(11):e0309883. doi: 10.1371/journal.pone.0309883 (PMC11548840; doi:10.1371/journal.pone.0309883)
Supplement: S1 Appendix — a The I-CVI (Item content validity index: I-CVI) was calculated by asking respondents to rate the degree to which each question item was related to the concept on a 4-point scale (1: not related to 4: fairly related), and the percentage of the number of respondents who answered 3 or 4 for each question was calculated. The first survey was 8 people rated the items, so items with an I-CVI of 0.75 or less were considered for modification. (DOCX) [file pone.0309883.s002.docx]

S1 Appendix.

Draft Questions Generated by Cognitive Interview and I-CVI Results (Round 1)

|  | | n=8 |
| --- | --- | --- |
| No. | items | I-CVI^a^ |
| 1 | If I were a staff receiving discharged patients, I wonder how I would feel knowing the care that has been provided to my patients in the hospital. | 0.88 |
| 2 | If I were a staff receiving discharged patients, I wonder what kind of care would I want to provide after the patient has been admitted from hospital. | 0.75 |
| 3 | If I were a staff receiving discharged patients, I wonder what kind of discharge support would I want for my patient prior to discharge. | 0.75 |
| 4 | If I were a staff receiving discharged patients, I wonder what would I want to discuss with the hospital nurse during the pre-discharge conference. | 0.88 |
| 5 | If I were the staff receiving discharged patients, I imagine how I would feel about any last minute changes to the discharge planning. | 0.25 |
| 6 | Whenever I have a disagreement with the staff receiving discharged patients about discharge support, I try to think about how I would feel if I were in the other person's shoes. | 0.75 |
| 7 | I consider whether a discharged patient is a patient who needs special attention from the staff receiving discharged patients. | 0.63 |
| 8 | I think about what patient information the staff receiving discharged patients requires. | 1.00 |
| 9 | I think about what patient family information the staff receiving discharged patients would require. | 1.00 |
| 10 | I think about how the information we provide to the staff receiving discharged patients will be understood by them. | 0.75 |
| 11 | I would consider whether the staff receiving discharged patients can implement care with the information they provide. | 0.88 |
| 12 | I would consider what is needed to make it easier for the staff receiving discharged patients to work with the hospital. | 0.75 |
| 13 | I think about how prepared the staff receiving discharged patients needs to be to facilitate care. | 0.88 |
| 14 | I would consider what adjustments need to be made in case the staff receiving discharged patients is unavailable or out of response time. | 0.25 |
| 15 | I think about what information the staff receiving discharged patients needs to respond to changes in the patient's condition. | 0.63 |
| 16 | I think about what information the staff receiving discharged patients would want regarding their care. | 1.00 |
| 17 | I also try to look at the position and situation of the staff receiving discharged patients. | 0.50 |
| 18 | I try to imagine how the staff receiving discharged patients would rate the care we gave. | 0.50 |
| 19 | I try to understand what the staff receiving discharged patients wants from our care. | 0.50 |
| 20 | I am trying to understand what the staff receiving discharged patients wants from our discharge support. | 0.75 |
| 21 | I try to imagine how the staff receiving discharged patients would be affected by a last minute change in the discharge planning document. | 0.38 |
| 22 | I think about the care that the staff receiving discharged patients must provide. | 0.88 |
| 23 | I consider the staffing situation of the staff receiving discharged patients. | 0.38 |
| 24 | I consider the amount of care that the staff receiving discharged patients can handle. | 0.63 |
| 25 | I would consider the skill level of the staff receiving discharged patients. | 0.25 |
| 26 | I would consider care items available to the staff receiving discharged patients | 0.50 |
| 27 | I think about the information that the staff receiving discharged patients has about the patient | 0.88 |
| 28 | I consider patient conditions that can be handled by the staff receiving discharged patients. | 0.63 |
| ^a^The I-CVI (Item content validity index: I-CVI) was calculated by asking respondents to rate the degree to which each question item was related to the concept on a 4-point scale (1: not related to 4: fairly related), and the percentage of the number of respondents who answered 3 or 4 for each question was calculated. The first survey was 8 people rated the items, so items with an I-CVI of 0.75 or less were considered for modification. | | |
