# Supplementary material for: Development of the Ward Nurses’ Perspective-taking of the Staff Receiving Discharged Patients Scale: An observational study of ward nurses
Source: PLoS One. 2024 Nov 8;19(11):e0309883. doi: 10.1371/journal.pone.0309883 (PMC11548840; doi:10.1371/journal.pone.0309883)
Supplement: S2 Appendix — Note: One nursing researcher dropped out from round 1. The order of the items was changed based on feedback from the initial cognitive interview. aThe I-CVI (Item content validity index: I-CVI) was calculated by asking respondents to rate the degree to which each question item was related to the concept on a 4-point scale (1: not related to 4: fairly related), and the percentage of the number of respondents who answered 3 or 4 for each question was calculated. The second round was 7 people rated the items, so items with an I-CVI of 0.83 or less were considered for modification. (DOCX) [file pone.0309883.s003.docx]

S2 Appendix.

Draft Questions Generated by Cognitive Interview and I-CVI Results (Round 2)

|  | | n=7 |
| --- | --- | --- |
| No. | items | I-CVI^a^ |
| 1 | I consider the role and situation of the staff receiving discharged patients. | 1.00 |
| 2 | I imagine what the staff receiving discharged patients would want from our discharge planning. | 1.00 |
| 3 | I think about the care that needs to be provided by the staff receiving discharged patients. | 1.00 |
| 4 | I think about the manpower of the facility receiving the discharged patients. | 0.83 |
| 5 | I think about the daily-living care that the staff receiving discharged patients might be able to handle. | 0.83 |
| 6 | I think about possible procedures and other medical techniques that can be performed by the staff receiving discharged patients. | 1.00 |
| 7 | I think about what care items are available at the site of the staff receiving discharged patients. | 1.00 |
| 8 | I think about what information the staff receiving discharged patients already has about the patient. | 0.83 |
| 9 | I consider whether the patient's condition can be handled by the staff receiving discharged patients. | 1.00 |
| 10 | I imagine how the staff receiving discharged patients would rate our care | 0.67 |
| 11 | I would consider what patient information I would require, if I were a staff receiving discharged patients. | 1.00 |
| 12 | I think about what patient family information I would need if I were the staff receiving discharged patients. | 0.83 |
| 13 | I think about what information I would need to manage a patient’s condition if I were the staff receiving discharged patients. | 1.00 |
| 14 | I think about how I would understand the information provided by the hospital where the receiving-patient was located if I were the staff receiving discharged patients. | 0.83 |
| 15 | I would consider whether I could implement care using information provided by the hospital where the patient is located, if I were a staff receiving discharged patients. | 1.00 |
| 16 | I imagine how I would feel knowing the care at the hospital where the patient located, if I were the staff receiving discharged patients. | 0.50 |
| 17 | I think about whether I would need to be careful when interacting with the patient if I were the staff receiving discharged patients. | 0.83 |
| 18 | I would think about what kinds of arrangements would make it easier to contact the hospital where the patient had been admitted after discharge from the hospital, if I were a staff receiving discharged patients. | 0.83 |
| 19 | I would consider what preparations are necessary at the hospital where the patient was located to make it easier to provide care after discharge, if I were a staff receiving discharged patients. | 0.83 |
| 20 | I would consider what information I want about their care, if I were the staff receiving discharged patients. | 1.00 |
| 21 | I imagine what care I would provide after receiving the patient, if I were a staff receiving discharged patients. | 1.00 |
| 22 | I imagine what adjustments I would want to see made at the hospital where the receiving patient was located, if I were a staff member at a facility that was unable to provide nursing care at night. | 0.83 |
| Note: One nursing researcher dropped out from round 1. The order of the items was changed based on feedback from the initial cognitive interview.  ^a^The I-CVI (Item content validity index: I-CVI) was calculated by asking respondents to rate the degree to which each question item was related to the concept on a 4-point scale (1: not related to 4: fairly related), and the percentage of the number of respondents who answered 3 or 4 for each question was calculated. The second round was 7 people rated the items, so items with an I-CVI of 0.83 or less were considered for modification. | | |
