# Supplementary material for: Development of the Ward Nurses’ Perspective-taking of the Staff Receiving Discharged Patients Scale: An observational study of ward nurses
Source: PLoS One. 2024 Nov 8;19(11):e0309883. doi: 10.1371/journal.pone.0309883 (PMC11548840; doi:10.1371/journal.pone.0309883)
Supplement: Attachment — Submitted filename: Response_to_Reviewers_Shingo_TANAKA.docx [file pone.0309883.s006.docx]

April 21, 2024

Fatma Ay

Academic Editor
PLOS ONE

Dear Dr. Ay:

Thank you for inviting us to submit our revised manuscript, “Development of the Ward Nurses’ Perspective-taking of the Staff Receiving Discharged Patients Scale: An Observational Study of Ward Nurses.”

We thank you and the reviewers for your thoughtful suggestions and insights. The manuscript has benefited from these insightful suggestions. I look forward to working with you and the reviewers to move this manuscript closer to publication in *PLOS ONE*.

We have incorporated a number of changes based on your suggestions, highlighted in yellow. We have also added new supplementary file based on the reviewer’s comments. To facilitate your review of our revisions, the following is a point-by-point response to the questions and comments in your letter dated April X, 2024.

Thank you for your consideration. I look forward to hearing from you.

Sincerely,

Shingo Tanaka

Department of Fundamentals of Nursing, School of Nursing, Yokohama City University, 3-9 Hukuura, Yokohama kanazawa-ku, Kanagawa 113-0033, Japan

Email: tanaka.4n5.res@gmail.com

Dear Editor

I made the following changes in order to adhere to the journal requirements:

1. I have reviewed my paper to check if it fits the PLOS One style.
2. I have added a title page into main manuscript.
3. The Acknowledgements section in title page has been changed as follows:

I would like to express my gratitude to all those who cooperated in the research.

Since no funding information is given in the main manuscript, I think there is no need to change the covering letter for now.

4. I have revised data availability statement.
5. I have verified that the ethics statements are not listed outside of the METHODS section.
6. I have included a separate caption and legend for each figure in my manuscript.
7. I have embedded tables into the main document.
8. I have added captions for supplementary files as an appendix.

[Reviewer(s)' Comments]

Reviewer #1

Thank you for your careful peer review. I have revised the manuscript based on your valuable suggestions. I will respond to each of your comments individually. The revised sections have been marked out in yellow highlight for your convenience.

1. This manuscript weakens the process of developing the scale, and only shows the verification results. The scale development process should be introduced in detail, and the scales obtained from each round of item selection, the items deleted and the final scale can be displayed in the appendix if there are too many contents.

Thank you for your comment. In line with your suggestion, I have now included the proposed items from the item creation process in the appendix. Additionally, an explanation regarding the process of scale development has been added. For details, please review my response to the comments from you, particularly those related to the method.

2. The exploratory factor analysis method is commonly employed in scale development. Could you please clarify why the manuscript utilized both exploratory and confirmatory factor analysis methods simultaneously? Kindly provide an explanation within the manuscript.

Thank you for pointing this out. In order to clarify, I have added the following explanation to the " Determination of factor structure” section:

This scale was designed with a multidimensional structure in mind. Therefore, EFA was employed to identify and name the factors from the item pool. Subsequently, CFA was conducted to verify the construct validity by examining whether the model fit statistically across different samples.

3. The author should review the usage of technical terminology throughout the entire text, such as replacing "patient transfer" with "patient referral".

Thank you for pointing this out. I have replaced the term "patient transfer" with “discharge planning” in the key word section. I have reviewed previous studies and recognize that discharge planning is a more appropriate term for this study, as described in the main document definition.

4. The full text format should be adjusted to align the two ends, enhancing its aesthetic appeal.

Thank you for advice. I have made the necessary formatting changes.

5. Keywords: The keywords should be separated by quotation marks, with each keyword capitalized at the beginning.

The keywords have now been capitalized and are separated by quotation marks as suggested..

6. Abstract: The abstract is excessively verbose. It is advisable to be more succinct.

Thank you for your advice, I have summarized the Introduction and Conclusion in the Abstract as follows:

**Introduction:** Discharge planning involves coordinating between different care settings. Failed coordination can lead to delayed care at the facilities receiving discharged patients. Nurses who implement discharge planning must consider the circumstances of the staff receiving the discharged patients.

**Conclusion:** The newly developed scale proved to be reliable, valid, and suitable for use. This scale can measure the degree of perspective-taking by nurses, which can improve collaboration between facilities and the effectiveness of discharge planning.

7. Introduction:
   a) The introduction is excessively lengthy. I recommend condensing it logically and merging relevant theoretical content, with a focus on elaborating the significance of nurses' perspective in receiving discharged patients for institutional staff.

Thank you for advice. In order to focus on perspective-taking, I have now condensed the paragraphs from the fifth paragraph onwards in the Introduction section.

b) “It is defined as ‘the active cognitive process of imagining the world from another’s vantage point or imagining oneself in another’s shoes to understand their visual viewpoint, thoughts, motivations, intentions, and/or emotions’ [12 pp. 94-95]”, the utilization of “12pp” in this sentence lacks standardization.

Thank you for pointing this out, I revised direct quotation style as following:

It is defined as “the active cognitive process of imagining the world from another’s vantage point or imagining oneself in another’s shoes to understand their visual viewpoint, thoughts, motivations, intentions, and/or emotions” [12] (94-95).

c) “In this study, the staff receiving discharged patients comprise nurses, home care nurses, and public health nurses in the jurisdictional district who receive patients from the hospital after discharge. ”，the paragraph commencing with this sentence introduces the purpose and significance within the preceding context, rendering it more suitable for placement in the methods section from both a content and structural standpoint.

Thanks for your suggestion. Based on the same, I have shifted the above text to the Method section as Definition.

8. Methods:
   a) In the section of “Data collection”, the three subheadings “Item development”, “Evaluation by an expert”, and “Scale validation” did not appear to fall within the scope of data collection. Change is recommended.

Thank you for suggestion. I have now replaced the “Data collection” subheading with “Three steps of item development.” Additionally, I have added in the “Data collection of scale validation” section to provide information on the online survey.

b) The section on “Evaluation by an expert” lacks clear inclusion and exclusion criteria for experts, a specific process for evaluating the scale, indicators for making modifications, and a presentation of the obtained scale evaluation in this study.

Thank you for pointing out. I have included the items of each round of cognitive interview and I-CVI as an appendix, and added following explanation in main document:

Eight participants were evaluated in the first round and seven in the second round (one nurse researcher dropped out).

and

The inclusion criteria are participants with clinical experiences of at least 5 years as a nurse and in discharge planning. The exclusion criteria are only having clinical experience in the maternity ward.

In the cognitive interviews, based on their clinical experience, participants were asked about the parts of the questions that were difficult to understand and ones that should be added.

c) The specific method of randomization used in the manuscript was not specified by the author, and no explanation of the process was provided. The authors are encouraged to provide a more detailed explanation of the randomization method employed and offer a comprehensive program description.

Thank you for pointing out. I have now added the following explanation under “surveyed facilities”:

Only acute-care hospitals were extracted from the hospital databases provided by each local health authority [32–34], and 200 facilities were requested to participate in the survey, and were assigned random numbers.

d) The section on "Surveyed facilities" lacks an explicit explanation in the text regarding the criteria and sources employed for selecting the 45 hospitals. It would be beneficial if the author could incorporate a comprehensive clarification within the text.

As mentioned above, I have included the following explanation under “surveyed facilities”:

Only acute-care hospitals were extracted from the hospital databases provided by each local health authority [32–34], and 200 facilities were requested to participate in the survey, and were assigned random numbers.

e) The “Participants” section should be organized and written in a manner that includes clear inclusion and exclusion criteria.

Thank you for advice. This section has been revised as follows:

The inclusion criteria are ward nurses who had engaged in discharge planning. The exclusion criteria are nurses with less than a year of nursing experience, did not work in wards, worked in obstetric wards and unit care such as intensive care units and emergency wards, and worked as head ward nursing managers.

9. Results:
   a) The low response rate of the questionnaire may result in sample underrepresentation, leading to bias and compromising the credibility of survey findings. With only 61 participants completing the second survey, it is necessary for the author to provide further explanations and clarifications in their manuscript.

Thank you for advice. From reviewing articles regarding discharge planning especially in Japan, I have determined that a response rate setting of about 20% is appropriate. As such, we have added the following explanations to the Method and Limitation sections, respectively:

(Method)

We performed sample size calculations to validate construct validity at time 1 and temporal stability at time 2. Regarding time 1, we found that 200 participants, 10 times the number of items in the draft scale, were needed based on the recommendation of Boateng et al. [22]. Regarding time 2, we found 19 participants were needed to calculate the intraclass correlation coefficients (ICC) between the first and the second surveys with 30 observed variables per participant (the null hypothesis was set at 0.5, the alternative hypothesis at 0.7, the power at 0.8, and the probability of significance at 0.05) [31]. The response rate was assumed to be about 20% based on previous discharge planning studies [32,33]. Therefore, it was determined that a distribution to approximately 1,000 persons was necessary for the analysis.

(Limitation)

The current study met the sample size requirements for time 1 and time 2 [22,31]. However, the response rate was low, similar to the response rate in a Japanese study of discharge planning. [32,33]. The low response rate may be due to the many questions and participants’ interest. In particular, a selection bias may have occurred if only nurses who expressed a strong interest in discharge planning were assumed to have responded to the study questions.

b) The ICC value is unsatisfactory, and it is recommended to discuss in the discussion section for an analysis of the underlying reasons as well as exploration of potential improvement methods.

Thank you for the suggestion. I have added the following sentence to the Discussion section in the discussion around the reason for the ICC being low.

Further studies should be conducted on 1) how nurses’ perspective-taking of staff receiving patients changes over time, 2) whether an educational effect was seen in responding to the scale twice, and for how long this effect remains on the nurses.

Furthermore, errors in the ICC and paired t-test values have been corrected.

c) The results section should include a demonstration of the reliability and validity of the scale development process.

Thanks you for pointing this out." I have now created subsections for "scale validity" and "scale reliability" in order to revise the paper based on your suggestion..

10. Discussion:
    a) Authors are advised to incorporate a concise summary of the findings section within the introductory paragraph of the discussion section. And insert it into the first paragraph of this section.

Thank you for advice. I added following sentences in first paragraph of Discussion section:

The EFA and CFA confirmed that the scale consists of two subfactors. Correlation analysis with other scales confirmed its construct validity, whereas Cronbach's alpha and ICC confirmed its reliability. However, the "imagine-self" subfactor was found to have low temporal stability.

b) As previously mentioned, this scale can assess the level of nurses' comprehension regarding the discharge plan staff, thereby enhancing inter-institutional collaboration and discharge plan effectiveness. The concept of perspective-taking originates from inherent human nature and falls within the realm of psychological research. While the impact of various motivations or intervention mechanisms may be more pronounced, this manuscript does not explicitly highlight the significance and value of this scale for discharge referral. Therefore, it is hoped that the author will provide an explanation in the discussion section.

Thank you for kind pointing out. I have now added following sentences in “scale availability” in Discussion section which serve as an explanation:

Prior research on nurses' perceptions focused on their interest in patients [20,21]. Additionally, studies on collaboration among medical staff focused on systems and the physical environment [54,55]. Therefore, the manner in which nurses' perceptions of the staff receiving discharged patients would affect their collaborations was unclear. The measures developed by this study focus on nurses’ recognition and contribute to clarifying the mechanism whereby the kind of event stimulates the perspective-taking and affects discharge planning.

c) "It differs from existing scales that measure the perspectives of other people and colleagues in daily life.", it lacks in-depth discussion on the comparison of scales, which measure the perspectives of other people and colleagues in daily life. I suggest providing specific explanations and conducting a thorough analysis to highlight the differences between this scale and existing ones, as well as exploring the underlying reasons for these distinctions.

Thank you for advice. I added following explanation to the “scale validity” sub-section in Discussion section:

It measures nurses’ perceptions of the resources available to the staff receiving discharged patients and the information they desire. Previous scales have measured perspective-taking toward friends in everyday life, staff in other departments of a company, and customers [11,17,18]. However, the scale developed in this study differs from other existing ones in that it measures perspective-taking toward staff in other facilities in the medical setting of discharge planning.

11. Acknowledgments: “This research was supported by XXX”, the expression is not precision enough.

Thank you for pointing this out. I have now revised the above as:

I would like to express my gratitude to all those who cooperated in the research.

12. Tables:
    a) In table 2, the specific explanation regarding F1 and F2 is currently unavailable.

Thank you for checking. I have revised the terms from F1 and F2 to “Factor 1” and “Factor 2” in Table 2.

b) The p-value annotation is not standardized and should be revised to p≤.

Thank you for your attention to detail. Accordingly, I have made the correction in Table 3.

Reviewer #2:

Thank you for your helpful comments and insightful suggestions. I have now revised the paper based on your advice. The revised sections have been marked out in yellow highlight for your convenience.

1. Line 97 to 103 should be part of the methods section. The last sentence of the Background section should be: "the aim of this study...."

Thanks for your suggestion. Based on the same, I have now shifted the above text to the Method section.

I have also revised the last sentence as follows:

Therefore, the aim of this study is to develop a scale to measure ward nurses’ perspective-taking of the staff receiving discharged patients (Ward Nurses’ Perspective-taking of the Staff Receiving Discharged Patients Scale ) and test the scale’s reliability and validity.

2. The limitations paragrahs require expansion, for example can a predominantly female sample also be valid for male nursing staff?

Thank you your insightful comment. Based on your suggestion, I have now added the following discussion in the Limitations section:

In the present study, the majority of the participants were female. It has been reported that gender does not affect perspective-taking [18]. Therefore, a bias due to gender is unlikely. However, perspective-taking is affected by the power relationship with the target [57,58] as well as differences between competitive and cooperative environments [16]. Therefore, future research should not only focus on the attributes of the participant's population, but also on the relationship between the perspective-taking target and participant population.

3. Is a response of 32-35% considered a high or acceptable response rate? In both cases of the questionnaire, time 1 and time 2, the response percentage was similar.

Thank you for advice. From reviewing articles regarding discharge planning especially in Japan, I have determined that a response rate setting of about 20% is appropriate. As such, we have added the following explanations to the Method and Limitation sections, respectively:

(Method)

The response rate was assumed to be about 20% based on previous discharge planning studies [32,33]. Therefore, it was determined that a distribution to approximately 1,000 persons was necessary for the analysis.

(Limitation)

The response rate for the current study was similar to that of previous studies on discharge planning especially in Japan [32,33]. However, the response rate for both Time 1 and Time 2 was low, at 30%. Therefore, the possibility of selection bias arises.

4. Line 164-165 Exclusion criteria mentioned - "nurse worked as nursing managers". Despite what is mentioned in the methods chapter, in table 1, it is written that about a third of the respondents to the first questionnaire are in management positions. Shouldn't they have been disqualified?

Thank you for pointing out. Nurses who meet the exclusion criteria are the top managers of the ward. The position in Table 1 is a nurse in the chief or team leader class. In order to clarify this, I have made the following revisions to the Method section and Table 1:

(Method)

The inclusion criteria are ward nurses who had engaged in discharge planning. The exclusion criteria are nurses with less than a year of nursing experience, did not work in wards, worked in obstetric wards and unit care such as intensive care units and emergency wards, and worked as head ward nursing managers.

(Table 1)

Deputy chief, Chief, Deputy head ward nursing manager
