# Supplementary material for: Development of the Ward Nurses’ Perspective-taking of the Staff Receiving Discharged Patients Scale: An observational study of ward nurses
Source: PLoS One. 2024 Nov 8;19(11):e0309883. doi: 10.1371/journal.pone.0309883 (PMC11548840; doi:10.1371/journal.pone.0309883)
Supplement: Attachment — Submitted filename: Second review report..docx [file pone.0309883.s007.docx]

**Comments to the Authors**

Thanks to the authors for showing us the revised manuscript of “Development of the Ward Nurses’ Perspective-taking of the Staff Receiving Discharged Patients Scale: An Observational Study of Ward Nurses”. Overall, this manuscript is interesting. But there are plenty of concerns.

1. Please further refine the screening process. Firstly, provide a comprehensive explanation in the manuscript regarding the rationale behind setting the items adjustment criteria at 0.75 and 0.83 (I-CVI). Secondly, if we consider using 0.75 as the screening criterion in the initial round, it would necessitate removing 13 items. Please explain how the 28 items reviewed in the first round were refined to 22 items reviewed in the second round. Also, how the final 20 items were determined.
2. **Keywords:** The format of keywords still contains some errors, which should be revised to a more standardized format: Hospital nursing staff; Multidisciplinary care team.
3. **Introduction:**

a) The introduction section remains excessively lengthy and it is advisable to streamline it further.

b) “It is defined as ‘the active cognitive process of imagining the world from another’s vantage point or imagining oneself in another’s shoes to understand their visual viewpoint, thoughts, motivations, intentions, and/or emotions’ [12] (94-95)”, please explain the specific meaning of “(94-95) ”and elucidate it within the manuscript.

**4. Methods:** In the section of “Surveyed facilities”, while the authors included details of	the randomization process for 200 hospitals, they did not clearly state the source of	involvement for 45 hospitals. It is necessary to clarify whether these 45 hospitals have any affiliation with the aforementioned 200 hospitals.
