# Supplementary material for: Development of the Ward Nurses’ Perspective-taking of the Staff Receiving Discharged Patients Scale: An observational study of ward nurses
Source: PLoS One. 2024 Nov 8;19(11):e0309883. doi: 10.1371/journal.pone.0309883 (PMC11548840; doi:10.1371/journal.pone.0309883)
Supplement: Attachment — Submitted filename: Response_to_Reviewers_Shingo_TANAKA.docx [file pone.0309883.s008.docx]

June 1, 2024

Fatma Ay

Academic Editor
*PLOS ONE*

Dear Dr. Ay:

Thank you for inviting us to submit our revised manuscript, “Development of the Ward Nurses’ Perspective-taking of the Staff Receiving Discharged Patients Scale: An observational study of ward nurses.”

We thank you and the reviewers for your thoughtful suggestions and insights. The manuscript has benefited from these insightful suggestions. I look forward to working with you and the reviewers to move this manuscript closer to publication in *PLOS ONE*.

We have incorporated a number of changes based on your suggestions, highlighted in yellow. To facilitate your review of our revisions, the following is a point-by-point response to the questions and comments in your letter dated May 24, 2024.

Thank you for your consideration. I look forward to hearing from you.

Sincerely,

Shingo Tanaka

Department of Fundamentals of Nursing, Nursing Course, School of Nursing, Yokohama City University, 3-9 Hukuura, Yokohama kanazawa-ku, Kanagawa 113-0033, Japan

Email: tanaka.4n5.res@gmail.com

[Reviewer(s)' Comments]

Reviewer #1

Thank you for your careful peer review. I have revised the manuscript based on your valuable suggestions. I will respond to each of your comments individually. The revised sections have been marked out in yellow highlight for your convenience.

Thanks to the authors for showing us the revised manuscript of “Development of the Ward Nurses’ Perspective-taking of the Staff Receiving Discharged Patients Scale: An Observational Study of Ward Nurses”. Overall, this manuscript is interesting. But there are plenty of concerns.

1. Please further refine the screening process. Firstly, provide a comprehensive explanation in the manuscript regarding the rationale behind setting the items adjustment criteria at 0.75 and 0.83 (I-CVI). Secondly, if we consider using 0.75 as the screening criterion in the initial round, it would necessitate removing 13 items. Please explain how the 28 items reviewed in the first round were refined to 22 items reviewed in the second round. Also, how the final 20items were determined.

**Response:** Thank you for pointing this out. The following explanations were added to address your comment:

The criteria for each round were calculated based on the recommendation of a previous study [29]. In both the rounds, items noted to have overlapping semantic content in the cognitive interviews were consolidated, and the use of subjects and predicates for each item was standardized. The researchers reviewed the items with I-CVI values below the criteria in the first round. Based on the results of the cognitive interviews, the items were rephrased, integrated with other items, or deleted, resulting in 22 items, down from the original 28. Items with I-CVI values that did not meet the criteria in the second round were similarly reviewed by the researchers and integrated into other items or deleted based on feedback from the cognitive interviews, resulting in 20 items from 22. The researchers reviewed the final draft to confirm the semantic content, and minor phrasing revisions were made to facilitate easy responses from participants.

2. Keywords: The format of keywords still contains some errors, which should be revised to a more standardized format: Hospital nursing staff; Multidisciplinary care team.

**Response:** Thank you for this suggestion. The format of the keywords has been revised to a standardized one. We checked with the guidelines and the editorial office and confirmed that there are no guidelines regarding keywords.

3. Introduction:
   a) The introduction section remains excessively lengthy and it is advisable to streamline it further.

**Response:** Thank you for the kind advise. We have reorganized the paragraph introducing prior research on discharge planning.

However, we believe that the paragraphs introducing perspective-taking, considerations on the scale, and the significance of the study are essential for explaining the subsequent development.

b) “It is defined as ‘the active cognitive process of imagining the world from another’s vantage point or imaginingoneself in another’s shoes to understand their visual viewpoint, thoughts, motivations, intentions, and/or emotions’[12] (94-95)”, please explain the specific meaning of “(94-95) ”and elucidate it within the manuscript.

**Response:** Thank you for this comment. (94-95) refers to the page numbers of the referenced publication. We have ensured that the in-text citation is formatted according to the Vancouver style.

4. Methods: In the section of “Surveyed facilities”, while the authors included details of the randomization process for200 hospitals, they did not clearly state the source of involvement for 45 hospitals. It is necessary to clarify whether these 45 hospitals have any affiliation with the aforementioned 200 hospitals.

**Response:** Thank you for pointing this out. We have revised the text to indicate that 200 facilities were randomly selected from the database, and the 45 facilities that cooperated were included in the survey.

Reviewer #2:

Thank you for your peer review. We have revised the manuscript in response to the other reviewer's comments. Please check our revised manuscript. We trust that the revisions have brought our manuscript closer to publication in *PLOS ONE.*
