# Supplementary material for: Development of the Ward Nurses’ Perspective-taking of the Staff Receiving Discharged Patients Scale: An observational study of ward nurses
Source: PLoS One. 2024 Nov 8;19(11):e0309883. doi: 10.1371/journal.pone.0309883 (PMC11548840; doi:10.1371/journal.pone.0309883)
Supplement: Attachment — Submitted filename: Third review report...docx [file pone.0309883.s009.docx]

**Comments to the Authors**

Thanks to the authors for showing us the revised manuscript of “Development of the Ward Nurses’ Perspective-taking of the Staff Receiving Discharged Patients Scale: An Observational Study of Ward Nurses”. Overall, this manuscript is much better now. But there are still some questions.

1. During the construction of the professional scale, these items should be deleted if items falls below the pre-set standard (I-CVI). If these items are to be reintegrated and rephrased with other items, it is necessary to conduct a survey of the scale again before experts evaluation can be carried out.
2. **Introduction:**
a) In the section of introduction, 3 to 5 paragraphs primarily show the current research situation about “perspective-taking”, which is excessively lengthy. It is recommended to streamline and summarize the theoretical aspects instead of presenting literature details.
b) The Vancouver style employs superscript numbering and rarely includes the page number of the referenced literature. "12 pp. 94-95" requires further correction, and the entire text should be standardized to a similar format: [1].
