# Supplementary material for: Development of the Ward Nurses’ Perspective-taking of the Staff Receiving Discharged Patients Scale: An observational study of ward nurses
Source: PLoS One. 2024 Nov 8;19(11):e0309883. doi: 10.1371/journal.pone.0309883 (PMC11548840; doi:10.1371/journal.pone.0309883)
Supplement: Attachment — Submitted filename: Response_to_Reviewers_Shingo_TANAKA.docx [file pone.0309883.s010.docx]

June 18, 2024

Fatma Ay

Academic Editor
*PLOS ONE*

Dear Dr. Ay:

Thank you for inviting us to submit our revised manuscript, “Development of the Ward Nurses’ Perspective-taking of the Staff Receiving Discharged Patients Scale: An observational study of ward nurses.”

We thank you and the reviewers for your thoughtful suggestions and insights. The manuscript has benefited from these insightful suggestions. I look forward to working with you and the reviewers to move this manuscript closer to publication in *PLOS ONE*.

We have incorporated a number of changes based on your suggestions, highlighted in yellow. To facilitate your review of our revisions, we have prepared and attached herewith a point-by-point response to the questions and comments in your letter dated June 12, 2024.

Thank you for your consideration. We look forward to hearing from you.

Sincerely,

Shingo Tanaka

Department of Fundamentals of Nursing, Nursing Course, School of Nursing, Yokohama City University, 3-9 Hukuura, Yokohama kanazawa-ku, Kanagawa 113-0033, Japan

Email: tanaka.4n5.res@gmail.com

[Editor Comments]

Please review your reference list to ensure that it is complete and correct. If you have cited papers that have been retracted, please include the rationale for doing so in the manuscript text, or remove these references and replace them with relevant current references. Any changes to the reference list should be mentioned in the rebuttal letter that accompanies your revised manuscript. If you need to cite a retracted article, indicate the article’s retracted status in the References list and also include a citation and full reference for the retraction notice.

**Response:** Thank you for raising this issue. We have updated the reference list in the revised manuscript. Additionally, we have checked whether each reference has been retracted by The Retraction Watch Database.

[Reviewer(s)' Comments]

Reviewer #1

Thank you for your careful peer review. We have revised the manuscript based on your valuable suggestions—the revised sections have been highlighted in yellow for your convenience. Furthermore, we have responded to each of your comments individually.

Thanks to the authors for showing us the revised manuscript of “Development of the Ward Nurses’ Perspective-taking of the Staff Receiving Discharged Patients Scale: An Observational Study of Ward Nurses”. Overall, this manuscript is much better now. But there are still some questions.

1. During the construction of the professional scale, these items should be deleted if items falls below the pre-set standard (I-CVI). If these items are to be reintegrated and rephrased with other items, it is necessary to conduct a survey of the scale again before experts evaluation can be carried out.

**Response:** Thank you for pointing this out. Based on previous research, in the first round, we did not remove items without due consideration. However, we included a process to create revised items from feedback. We have revised the section “Evaluation by experts” to convey this.

2. Introduction:
   a) In the section of introduction, 3 to 5 paragraphs primarily show the current research situation about “perspective-taking”, which is excessively lengthy. It is recommended to streamline and summarize the theoretical aspects instead of presenting literature details.

**Response:** Thank you for pointing this out. We have streamlined the Introduction based on your comment.

b) b) The Vancouver style employs superscript numbering and rarely includes the page number of the referenced literature. "12 pp. 94-95" requires further correction, and the entire text should be standardized to a similar format: [1].

**Response:** We agree with the reviewer’s advice and have revised the in-text citations and references as per the Vancouver style using numbers in square brackets according to the journal guidelines.

Reviewer #3:

It is a good topic for Nurse- mangers to apply this developed scale " Staff Receiving Discharged Patients Scale for Ward Nurses at hospitals.

The findings and results benefit all in-charge nurses to have a better plan to discharge the patients .

It is recommended to revise the whole paper by English language in Nursing sciences, It will be better.

**Response:** Thank you for pointing this out. We have had the revised manuscript checked by a professional proofreading service to ensure good language quality.
